# TNF-α Increases IP-10 Expression in MCF-7 Breast Cancer Cells via Activation of the JNK/c-Jun Pathways

**DOI:** 10.3390/biom11091355

**Published:** 2021-09-13

**Authors:** Shihab Kochumon, Amnah Al-Sayyar, Texy Jacob, Amal Hasan, Fahd Al-Mulla, Sardar Sindhu, Rasheed Ahmad

**Affiliations:** 1 Immunology & Microbiology Department, Dasman Diabetes Institute, Dasman 15462, Kuwait; shihab.kochumon@dasmaninstitute.org (S.K.); amnah.alsayyar@dasmaninstitute.org (A.A.-S.); texy.jacob@dasmaninstitute.org (T.J.); amal.hasan@dasmaninstitute.org (A.H.); sardar.sindhu@dasmaninstitute.org (S.S.); 2 Genetics & Bioinformatics Department, Dasman Diabetes Institute, Dasman 15462, Kuwait; fahd.almulla@dasmaninstitute.org; 3 Animal and Imaging Core Facility, Dasman Diabetes Institute, Dasman 15462, Kuwait

**Keywords:** TNF-α, IP-10/CXCL10, MCF-7, JNK, c-Jun

## Abstract

IP-10 (also called CXCL10) plays a significant role in leukocyte homing to inflamed tissues, and increased IP-10 levels are associated with the pathologies of various inflammatory disorders, including type 2 diabetes, atherosclerosis, and cancer. TNF-α is a potent activator of immune cells and induces inflammatory cytokine expression in these cells. However, it is unclear whether TNF-α is able to induce IP-10 expression in MCF-7 breast cancer cells. We therefore determined IP-10 expression in TNF-α-treated MCF-7 cells and investigated the mechanism involved. Our data show that TNF-α induced/upregulated the IP-10 expression at both mRNA and protein levels in MCF-7 cells. Inhibition of JNK (SP600125) significantly suppressed the TNF-α-induced IP-10 in MCF-7 cells, while the inhibition of p38 MAPK (SB203580), MEK1/2 (U0126), and ERK1/2 (PD98059) had no significant effect. Furthermore, TNF-α-induced IP-10 expression was abolished in MCF-7 cells deficient in JNK. Similar results were obtained using MCF-7 cells deficient in c-Jun. Moreover, the JNK kinase inhibitor markedly reduced the TNF-α-induced JNK and c-Jun phosphorylation. The kinase activity of JNK induced by TNF-α stimulation of MCF-7 cells was significantly inhibited by SP600125. Altogether, our novel findings provide the evidence that TNF-α induces IP-10 expression in MCF-7 breast cancer cells via activation of the JNK/c-Jun signaling pathway.

## 1. Introduction

The breast tumor microenvironment is one of the crucial elements supporting breast cancer tumor progression and metastasis [1]. Chemokines mediate immune cells trafficking into the tumor microenvironment and aggravate inflammation, which associates with the pathological responses [2]. Chemokines are soluble, small molecular weight (8–14 kDa) immunoregulatory proteins that are essential for immune cell chemotaxis and play a key role in a wide variety of contexts, including inflammation, host defense, apoptosis, cell growth and proliferation, angiogenesis, hematopoiesis, embryogenesis, lymphoid development, wound healing, tumorigenesis, cancer immunoediting, and metastasis [3]. IP-10, or CXCL10, is a small (8.7 kDa) proinflammatory chemokine that belongs to the CXC chemokine family and was originally identified and reported by Luster et al. in interferon (IFN)-γ-treated monocytic cells [4]. IP-10 is involved in regulation of immune responses through the recruitment and activation of leukocytes and endothelial cells [5] as well as other CXCR3+ cell types, including activated CD4+ T cells (Th1 subset), CD8+ T cells, natural killer (NK) cells, macrophages, plasmacytoid dendritic cells (DCs), and B cells [6]. It is highly expressed in various inflammatory diseases, including type 2 diabetes, cardiovascular disease, atherosclerosis, rheumatoid arthritis, multiple sclerosis, pulmonary fibrosis, liver injury, neurodegenerative diseases, and cancer [7,8,9,10]. Increased circulatory levels of IP-10 have been also documented in metabolic inflammatory conditions, such as obesity and type-2 diabetes mellitus (T2DM), and elevated IP-10 levels in patients with T2DM were related with insulin resistance [11]. IP-10 is known to have both angiogenic and angiostatic (anti-angiogenic) activities, and importantly, the balance between angiogenic and angiostatic factors controlled by bioactive molecules involved in inflammatory processes may impact the pathogenesis of many diseases [12,13].

IP-10 is also known to be a pro-tumorigenic chemokine that has been strongly correlated with the progression of breast cancer given that it is more largely secreted by the malignant than non-malignant tissues [14]. IP-10 is produced by multiple cell types, such as activated monocytes, macrophages, neutrophils, DCs, pre-adipocytes/adipocytes, hepatocytes, astrocytes, endothelial cells, keratinocytes, fibroblasts, mesenchymal cells, and pancreatic β cells [9,15,16,17] as well as by breast tumor cells, per se [18]. IP-10 expression is induced by lipopolysaccharide and proinflammatory cytokines, including IL-1 and tumor necrosis factor-α (TNFα) [19,20]. Increased circulating levels of both TNFα and IP-10 have been found in inflammatory disorders and multiple cancers [20,21]. However, the role of TNF-α in IP-10 production by MCP-7 breast tumor cells is not yet elucidated. Herein, we determined whether TNF-α could induce IP-10 production in metastatic breast cancer MCF-7 cells and if so, which signal transduction pathway(s) were involved in TNFα-driven IP-10 induction. Our data show that TNF-α induces the expression of IP-10 in MCF-7 cells. Moreover, pharmacologic and genetic inhibition of JNK suppressed the TNFα-induced IP-10 expression in MCF-7 cells. We further demonstrate that JNK phosphorylation was induced by TNF-α in MCF-7 cells. The kinase assay of JNK in MCF-7 cells confirmed its stimulation by TNF-α. The JNK inhibitor, SP600125, markedly suppressed the TNF-α-stimulated JNK phosphorylation and kinase activity, indicating the involvement of JNK/c-Jun in IP-10 gene expression in MCF-7 cells.

## 2. Materials and Methods

### 2.1. Cell Culture

MCF-7 cell line is a well-established model for cancer research related to gene regulation. Human MCF-7 cells were purchased from the American Type Culture Collection (ATCC, Manassas, VA, USA). For cell culture assays, cells were grown in MEM culture medium (Gibco, Thermo Fisher Scientific, Waltham, MA, USA) supplemented with 10% fetal bovine serum (Gibco, Thermo Fisher Scientific, Waltham, MA, USA), 2 mM glutamine (Gibco, Thermo Fisher Scientific, Waltham, MA, USA), 1 mM sodium pyruvate, 10 mM HEPES, 50 U/mL penicillin, and 50 μg/mL streptomycin (P/S; Gibco, Thermo Fisher Scientific, Waltham, MA, USA) and incubated at 37 °C (with humidity) in 5% CO_2_.

### 2.2. Cell Stimulation

MCF-7 cells were plated in 12-well plates (Costar, Corning Incorporated, Corning, NY, USA) at a cell density of 0.25 × 10^6^ cells/well, unless otherwise indicated. After 48 h incubation, cells were treated with vehicle or TNF-α (20 ng/mL; 210-TA, R&D Systems, Minneapolis, MN, USA) for 24 h at 37 °C. Cells were harvested for total RNA isolation, and conditioned media were collected for measuring the secreted IP-10 protein.

Regarding MAPK/JNK/c-Jun signaling pathway analysis, cells were preincubated under above-mentioned culture conditions for 1 h using different cell signaling pathway inhibitors, including the p38 MAPK inhibitor SB203580 (10 µM/mL; 559389, Sigma-Aldrich, Merck KGaA, Darmstadt, Germany), ERK1/2 inhibitor PD98059 (10 µM/mL; tlrl-pd98, InvivoGen, San Diego, CA, USA), MEK1/2 inhibitor U0126 (10 µM/mL; tlrl-u0126, InvivoGen, San Diego, CA, USA), and JNK inhibitor SP600125 (10 µM/mL; 420119, Sigma-Aldrich, Merck KGaA, Darmstadt, Germany), followed by cell stimulation with TNF-α for 24 h.

### 2.3. Real-Time Quantitative PCR

Total RNA was extracted from MCF-7 cells using RNeasy Mini Kit (Qiagen, Valencia, CA, USA) as per the manufacturer’s instructions. The cDNA was synthesized using 1 μg of total RNA using a high-capacity cDNA reverse transcription kit (Applied Biosystems, Foster City, CA, USA). Real-time PCR was performed on a 7500 Fast Real-Time PCR System (Applied Biosystems, Foster City, CA, USA) using a TaqManTM Gene Expression Master Mix (Applied Biosystems, Foster City, CA, USA) [22,23,24]. Each reaction contained 50 ng cDNA that was amplified with Inventoried TaqMan Gene Expression Assay products (IP-10, Hs00171042_m1; c-Jun, Hs01103582_s1; JNK, Hs01548508_m1; and GAPDH, Hs03929097_g1). Threshold cycle (Ct) values were normalized to house-keeping gene (GAPDH) expression, and the amounts of target mRNA relative to control were calculated using the ΔΔCt (2^–∆∆Ct^) method [25]. Relative mRNA expression was expressed as fold expression over average of control gene expression. The expression level in the control treatment was assumed to be 1 [26,27,28]. 

### 2.4. Small Interfering RNA (siRNA) Transfections

Transient transfection of MCF-7 cells were done using Lipofectamine RNAiMAX reagent (Thermo Fischer, USA) following the manufacturer’s instructions, and cells were transfected separately with siRNA-JNK (20 nM; s1320, Thermo Fischer, USA), c-Jun siRNA (20 nM; s7660, Thermo Fischer, MD, USA) and scrambled (control) siRNA (20 nM; Thermo Fischer, USA) [29,30]. After 48 h of transfection, cells were treated with TNF-α and incubated for 24 h. Cells and conditioned media were harvested for RNA isolation and ELISA. JNK and c-Jun gene knockdown levels were assessed by real-time qPCR using genes-specific primer probes as described above.

### 2.5. IP-10 Protein Determination in Cell Supernatants

Secreted IP-10 protein was quantified in supernatants of MCF-7 cells stimulated with TNFα using Human CXCL10/IP-10 Quantikine ELISA Kit (DIP-100, R&D Systems, Minneapolis, MN, USA), following the manufacturer’s instructions.

### 2.6. Western Blotting

MCF-7 cells treated with TNF-α for 15 min were harvested and incubated for 30 min with lysis buffer (10X Lysis Buffer, Cell Signaling, Danvers, MA, USA). The lysates were centrifuged at 14,000× *g* for 10 min for clarification, and supernatants containing cellular proteins were collected. Protein concentration was measured by Quickstart Bradford Dye Reagent, 1x Protein Assay kit (Bio-Rad Laboratories, Inc, CA) [31,32]. Protein (20 μg) samples were mixed with loading buffer, heated for 5 min at 95 °C, and resolved by 12% SDS-PAGE as described earlier [25,33] Cellular proteins were transferred to an Immuno-Blot Polyvinylidene difluoride (PVDF) membrane (Bio-Rad Laboratories, Hercules, CA, USA) by electroblotting. The membranes were then blocked with 5% non-fat milk in PBS for 1 h, followed by incubation with primary antibodies against p-SAPK/JNK (cat# 9251), SAPK/JNK (cat#9252), p-c-Jun (cat# 9261), and c-Jun (cat# 9165) in 1:1000 dilution at 4 °C overnight. All primary antibodies were purchased from Cell Signaling (Cell Signaling Technology Inc., Danvers, MA, USA). The blots were then washed three times with TBS-T and incubated for 2 h with HRP-conjugated secondary antibody (Promega, Madison, WI, USA). Immunoreactive bands were developed using an Amersham ECL Plus Western Blotting Detection System (GE Healthcare, Chicago, IL, USA) and visualized by Molecular Imager^®^ VersaDocTM MP Imaging Systems (Bio-Rad Laboratories, Hercules, CA, USA) [33].

### 2.7. Immunocytofluorescence

MCF-7 cells were seeded on coverslips and cultured in 12 well plates at 37  °C. After treatment and incubation, slides were fixed in 4% formaldehyde and washed three times in cold PBS. Cells were then permeabilized in 0.1% Triton X-100, followed by three washes in cold PBS. The samples were blocked in 1% bovine serum albumin for 1 h. The slides were incubated overnight at room temperature with the primary antibody, which was rabbit polyclonal anti-IP-10 antibody (R&D, USA) in 1:200 dilution. The cells were then washed in PBS containing 0.05% Tween three times and incubated for 1 h at room temperature with the secondary antibody, which was goat anti-rabbit IgG conjugated with Alexa Fluor 488 (abcam^®^ ab150077) in 1:200 dilution. After washing several times in PBS as before, cells were counterstained and mounted with coverslip using mountant containing DAPI (Vectashield H1500, Vectorlab, Burlingame, CA, USA). Confocal images of MCF-7 cells were collected on an inverted Zeiss LSM710 AxioObserver microscope (Carl Zeiss, Gottingen, Germany) using a Plan-Apochromat 40×/1.40 oil DIC M27 objective lens. Excitation was via a 647 nm HeNe solid-state laser and 405 nm line of an argon ion laser. After laser excitation of the samples, optimized emission-detection bandwidths were configured using Zeiss Zen 2012 control software (version 1.1.1.2.0, Gottingen, Germany). Subsequently, confocal images were captured, and fluorescence was measured using Zeiss Zen 2012 software (Gottingen, Germany) [34].

### 2.8. SAPK/JNK Kinase Assay

SAPK/JNK kinase activity was assessed using SAPK/JNK Kinase assay kit (Cell Signaling, USA) according to the manufacturer’s instructions. Briefly, cell lysates were prepared in lysis buffer using MCF-7 cells treated with TNF-α and vehicle-treated controls. Immunoprecipitation of phospho SAPK/JNK was done using immobilized phospho-SAPK/JNK rabbit mAb linked to agarose beads to pull down SAPK/JNK kinases from cell extracts. The immunoprecipitated SAPK/JNK extract was incubated with c-Jun fusion protein, kinase buffer, and ATP. Next, c-Jun phosphorylation mediated by SAPK/JNK kinase was measured by immunoblotting using phospho c-Jun antibody.

### 2.9. Statistical Analysis

Statistical analysis was performed using GraphPad Prism software (version 6.07, La Jolla, CA, USA). Data are shown as mean ± standard error of the mean (SEM), unless otherwise indicated. Student’s *t*-test and one-way ANOVA followed by Tukey’s test were used to compare means between groups. For all analyses, data from a minimum of three sample sets were used for statistical calculation. All *p*-values < 0.05 were considered significant (ns, non-significant, * *p* < 0.05, ** *p* < 0.01, *** *p* < 0.001, and **** *p* < 0.0001).

## 3. Results

### 3.1. TNF-α Treatment Upregulates IP-10 Gene and Protein Expression in MCF-7 Cells 

**Figure 1:**
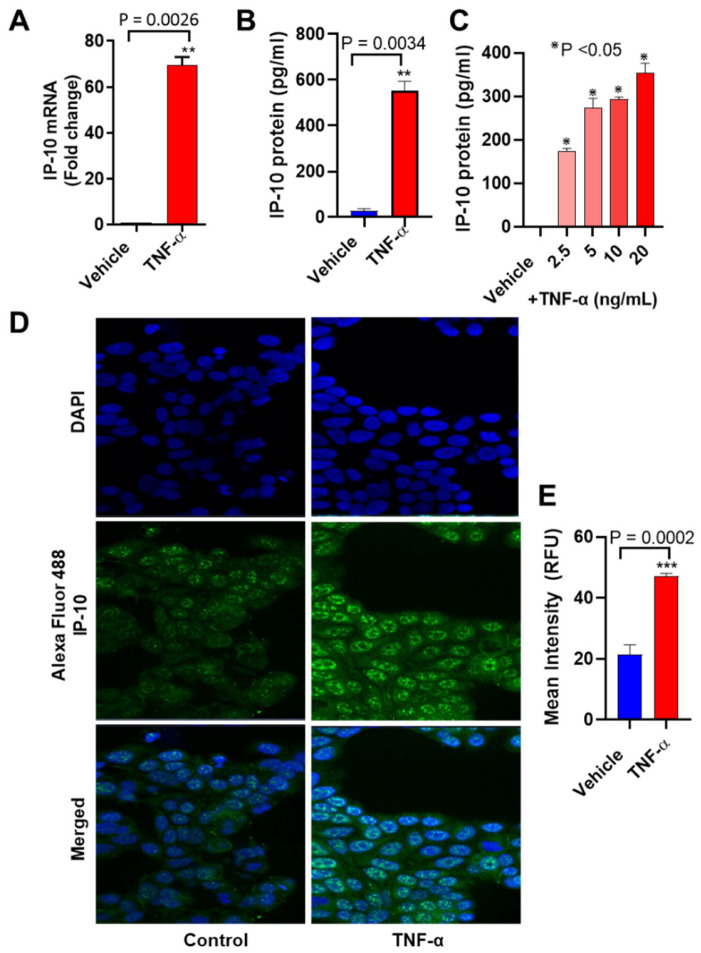
Effect of TNF-α on IP-10 production in human MCF-7 cells. MCF-7 cells were cultured in 12-well plates at a concentration of 0.25 × 10^6^ cells/well. Cells were treated separately with vehicle and TNF-α (20 ng/mL) for 24 h. After 24 h incubation, cells and supernatants were collected. (**A**) Total cellular RNA was isolated, and *IP-10* mRNA expression was determined by real-time RT-PCR. (**B**) Secreted IP-10 protein in culture media was determined by ELISA. (**C**) MCF-7 cells were treated with different concentrations of TNF-α (2.5, 5, 10, and 20 ng/mL) for 24 h, and protein expression of IP-10 was determined and compared in conditioned media. (**D**) MCF-7 cells treated with vehicle and TNF-α for 24 h were stained for confocal microscopy, as described in Materials and Methods. IP-10 expression is shown by green fluorescence (Alexa Flour 488), whereas nuclei are stained blue with DAPI (original magnification: 40×). (**E**) Relative fluorescence intensity of IP-10 was determined from random regions of the images. All data are expressed as mean ± SEM (*n* = 3). * *p* < 0.05, ** *p* < 0.01, *** *p* < 0.001.

TNF-α is a potent activator of immune cells and induces inflammatory cytokine expression, including IP-10 [20,35,36]. Since IP-10 induction by TNF-α in breast tumor cells has yet not been studied, we sought to determine the impact of TNF-α stimulation on the regulation of IP-10 expression in MCF-7 cells. To investigate whether TNF-α could induce/upregulate *IP-10* gene expression, we treated MCF-7 cells with TNF-α for 24 h, and both cells and conditioned media were harvested. *IP-10* gene expression in cells was determined by real-time RT-PCR using total cellular mRNA, and secreted IP-10 protein in conditioned media was measured by ELISA. Our data show that *IP-10* mRNA expression levels were significantly higher (69-fold; *p* = 0.002) in TNF-α-treated MCF-7 cells than those of controls (cells treated with vehicle only) (Figure 1A). Concordantly, IP-10-soluble protein levels were also significantly higher in MCF-7 cell supernatants after stimulation with TNF-α (523 ± 30 pg/mL; *p* = 0.003) (Figure 1B). Moreover, the effect of TNF-α on IP-10 secretion was dose dependent (Figure 1C). In addition, elevated IP-10 protein expression in MCF-7 cells was further confirmed by confocal microscopy (green fluorescence; Figure 1D,E).

### 3.2. JNK Inhibition Attenuates the TNFα-Induced IP-10 Production

**Figure 2:**
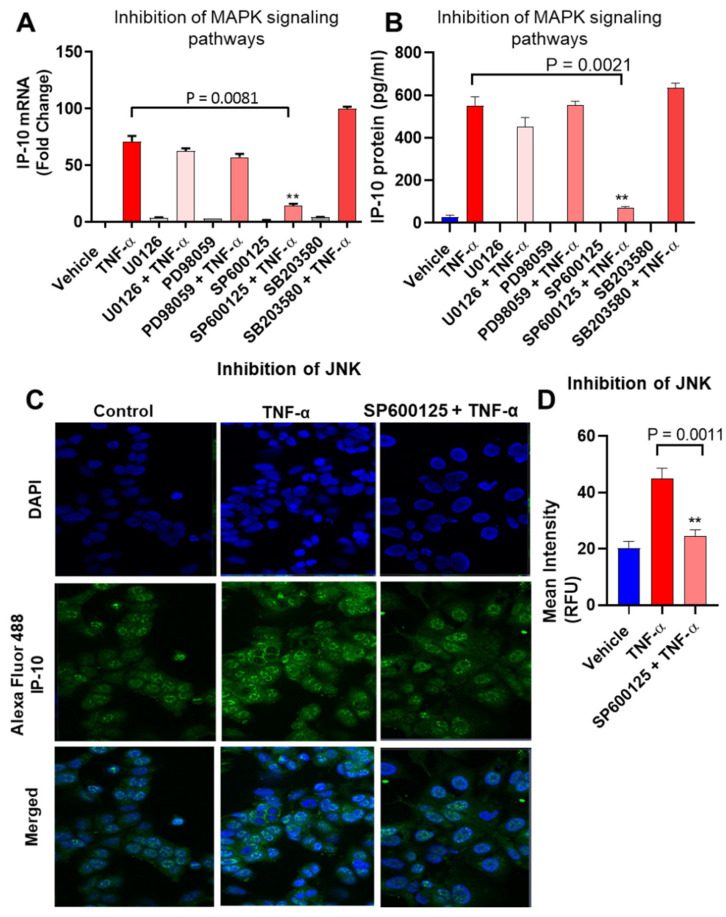
TNF-α-induced IP-10 expression is suppressed by inhibiting the JNK signaling pathway. (**A**,**B**). MCF-7 cells were pretreated for 1 h with inhibitors of MEK1/2 (U0126; 10 µM), ERK1/2 (PD98059; µM), JNK (SP600125; 10 µM), and p38 (SB203580; 10 µM) and then incubated with TNF-α for 24 hrs. Cells and culture media were collected. IP-10 mRNA and protein expression were determined using real-time RT-PCR and ELISA, respectively. (**C**) MCF-7cells were pretreated with SP600125 (10 µM) for 1 h, followed by TNF-α (20 ng/mL) treatment for 24 h. MCF-7 cells were then immune-stained for confocal microscopy, as described in Materials and Methods. IP-10 expression is shown by green fluorescence (Alexa Flour 488), whereas nuclei are stained blue with DAPI (original magnification 40×). (**D**) Relative fluorescence intensity of IP-10 expression was determined from random regions of the images. All data are expressed as mean ± SEM (*n* = 3). ** *p* < 0.01.

The p38, JNK, and ERK MAPK kinases are known to be regulated in different cell types by exposure to proinflammatory cytokine TNF-α [37,38]. To determine whether these kinases were involved in the TNF-α-induced upregulation of IP-10, MCF-7 cells were pretreated with inhibitors of MEK1/2 (U0126), ERK1/2 (PD98059), JNK (SP600125), and p38 (SB203580), followed by cell stimulation with TNF-α. Our data show that inhibition of the JNK signaling pathway significantly suppressed both the gene expression and protein secretion of IP-10 in MCF-7 cells in response to TNF-α stimulation, whereas inhibition of the p38, ERK1/2, and MEK1/2 signaling had a non-significant effect in these cells (Figure 2A,B). In MCF-7 cells, the suppression of IP-10 expression by inhibition of JNK was also confirmed by confocal microscopy (green fluorescence; Figure 2C,D). Next, we asked whether TNF-α-induced IP-10 gene expression and protein secretion in MDA-MB 231 breast cancer cells were suppressed by similar pathways as were seen in MCF-7 breast cancer cells. We treated MDA-MB 231 breast cancer cells with MAPKs inhibitors and then incubated with TNF-α. Our results show that inhibition of MAPK pathways suppressed TNF-α-induced IP-10 gene expression and protein secretion in MDA-MB 231 breast cancer cells (Supplementary Figure S1). However, p38 inhibition plays a predominant role in suppressing TNF-α-mediated IP-10 expression. We also found that inhibition of NF-kB suppressed TNF-α induced IP-10 in MCF-7 cells (Supplementary Figure S2).

### 3.3. JNK Deficiency Suppresses the TNF-α-Induced IP-10 Expression

**Figure 3:**
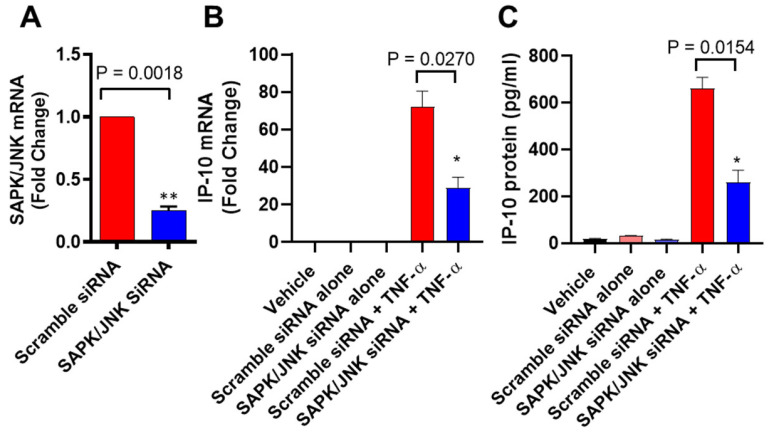
TNF-α-induced IP-10 expression is attenuated in JNK-deficient cells. MCF-7 cells were transfected with either JNK siRNA (20 nM) or scrambled siRNA (20 nM), as described in Materials and Methods. (**A**) After 36 h, real-time RT-PCR was performed to measure *JNK* mRNA expression. (**B**,**C**) MCF-7 sells transfected with JNK siRNA or scrambled siRNA were incubated with TNF-α (20 ng/mL) for 24 h. Cells and supernatants were collected, and IP-10 mRNA and protein were measured by real-time RT-PCR and ELISA, respectively. All data are expressed as mean ± SEM (*n* = 3). * *p* < 0.05, ** *p* < 0.01.

To verify that the induction of IP-10 by TNF-α was dependent on JNK signaling, we transfected MCF-7 cells with JNK siRNA, which resulted in a significant reduction in *JNK* mRNA expression levels compared to scrambled siRNA control (Figure 3A). Our data showed that siRNA-mediated knockdown of JNK significantly reduced the TNF-α-induced IP-10 expression (Figure 3B,C), confirming that induction of IP-10 by TNF-α requires JNK-mediated signaling.

### 3.4. c-Jun Deficiency Suppresses the TNF-α-Induced IP-10 Expression

**Figure 4:**
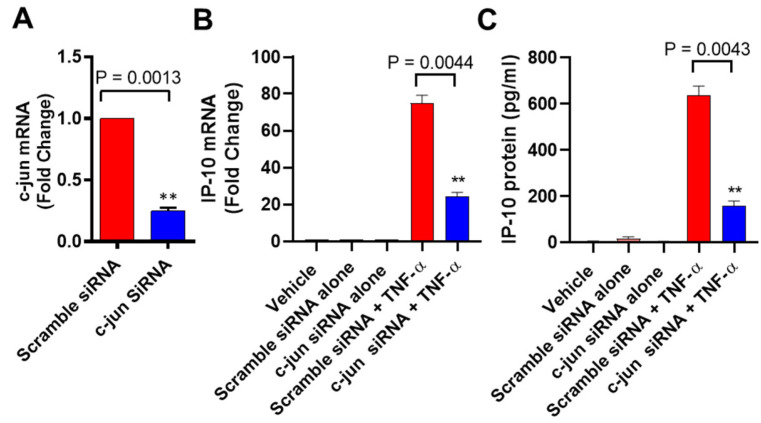
TNF-α-induced IP-10 expression is attenuated in c-Jun-deficient cells. MCF-7 cells were transfected separately with c-Jun siRNA (20 nM) and scrambled siRNA (20 nM), as described in Materials and Methods. (**A**) After 36 h, real-time RT-PCR was performed to measure c-Jun expression. (**B**,**C**) MCF-7 cells transfected with c-Jun siRNA and scrambled siRNA were incubated with TNF-α for 24 h. Cells and supernatants were collected, and IP-10 mRNA and protein expression were determined by real-time RT-PCR and ELISA, respectively. All data are expressed as mean ± SEM (*n* = 3). ** *p* < 0.01.

Given that c-Jun is located downstream of JNK, loss of c-Jun is expected to reduce the TNF-α-induced IP-10 expression in MCF-7 cells. To verify that the induction of IP-10 by TNF-α was dependent on c-Jun signaling, we transfected cells with c-Jun siRNA which resulted in a significant reduction in *c-Jun* mRNA expression levels compared to scrambled siRNA control (Figure 4A). Our data show that siRNA-mediated knockdown of c-Jun led to the same result as seen in case of JNK knockdown (Figure 4B,C), confirming that the induction of IP-10 by TNF-α requires c-Jun kinase signaling.

### 3.5. Effect of the TNF-α Stimulation on the Kinase Activity of JNK in MCF-7 Cells

**Figure 5:**
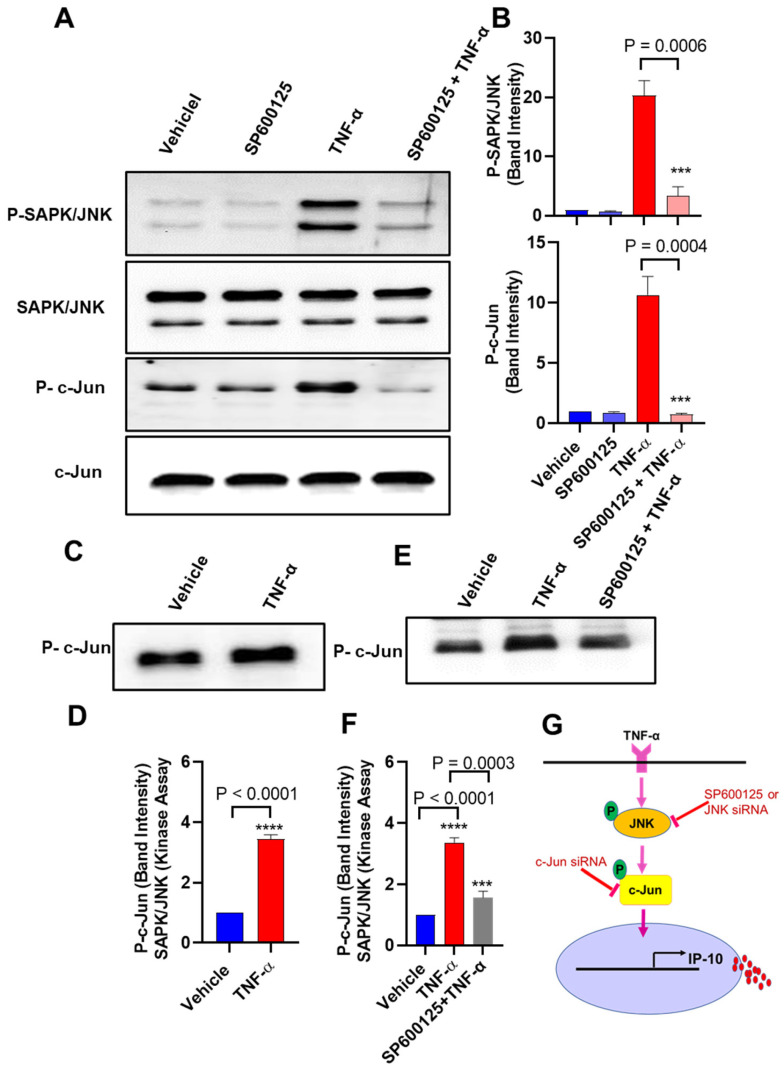
TNF-α induces IP-10 expression in MCF-7 cells through the JNK- and c-Jun-mediated signaling. (**A**) MCF-7 cells were pretreated with SAPK/JNK inhibitor (SP600125: 20 μM) for 1 h and then stimulated with TNF-α (20 ng/mL) for 15 min. Cell lysates were prepared and run on denaturing gel, as described in Materials and Methods. Phosphorylated SAPK/JNK and c-Jun are depicted in the upper panels, and total respective proteins are shown in the lower panels. (**B**) Densitometry data were used to calculate the phosphorylated to total protein ratios relative to control. (**C**) SAPK/JNK kinase assay was performed using lysates prepared from the TNF-α-treated and vehicle-treated MCF-7 cells, as described in Materials and Methods. (**D**) Band densitometry analysis relative to control shows significant upregulation of SAPK/JNK kinase activity/ c-Jun phosphorylation in TNF-α-treated cells compared to vehicle-treated controls. (**E**) SAPK/JNK kinase assay was performed using lysates from MCF-7 cells pretreated with SAPK /JNK inhibitor (SP600125; 20 µM) for 1 h, followed by TNF-α (20 ng/mL) treatment for 15 min. (**F**) Band densitometric analysis relative to control shows significant reduction in the level of SAPK/JNK kinase activity/ c-Jun phosphorylation in cells pretreated with SAPK/JNK inhibitor compared to TNF-α-alone treated cells. (**G**) Schematic illustration of signaling pathway underlying TNF-α-induced expression of IP-10. All data are expressed as mean ± SEM (*n* = 3). *** *p* < 0.001, **** *p* < 0.0001.

Next, we determined TNF-α-mediated phosphorylation and activation of JNK in MCF-7 cells. Our results that JNK and c-Jun phosphorylation was induced in MCF-7 cells, following stimulation by TNF-α. MCF-7 cells pretreated with SP600125 inhibitor showed a significant decrease in the TNF-α-induced JNK and c-Jun phosphorylation (Figure 5A,B). We further examined the effect of TNF-α stimulation on the kinase activity of JNK. To this end, lysates of MCF-7 cells were incubated overnight with 20 µL of c-Jun fusion protein beads, and then a kinase assay was performed. Results show that the kinase activity of JNK in TNF-α-stimulated MCF-7 lysates was significantly upregulated compared to that in control lysates (Figure 5C,D), while the pre-incubation with SP600125 inhibitor significantly suppressed TNF-α-induced JNK kinase activity (Figure 5E,F).

Schematic illustration of signaling pathway underlying TNF-α-induced expression of IP-10 (Figure 5G).

## 4. Discussion

Breast cancer occurs worldwide in women at any age following puberty, and the prevalence rates are higher in later life. According to the World Health Organization, by the end of 2020, about 7.8 million women alive had been diagnosed with breast cancer in the past five years, making it the most prevalent cancer worldwide. In this study, we report, for the first time to our knowledge, that TNF-α induces expression of IP-10 in MCF-7 breast carcinoma cells. Breast cancer cells are known to be highly secretory by nature, and their secretory activity can be modulated by a variety of inflammatory stimuli found in the tumor microenvironment. TNF-α is one of the key proinflammatory cytokines that are found in breast tumor microenvironment, and it is secreted by tumor-infiltrating macrophages, stromal cells, and also by tumor cells themselves. TNF-α plays a pro-tumorigenic role during the breast cancer progression and metastasis [39]. Increased serum TNF-α levels in stages II and III patients were associated with breast cancer cell invasiveness and poor prognosis, while there was a strong association between breast cancer prognosis and the expression levels of estrogen receptor (ER), progesterone receptor (PR), and human epidermal growth factor receptor (HER)-2/neu [40]. Our finding that TNF-α per se induces IP-10 in breast cancer cells is noteworthy since IP-10 is a potent leukocyte chemoattractant for activated monocytes/macrophages, T cells, and dendritic cells. Its presence can also impact the tumor microenvironment via effects on cell growth and proliferation, angiogenesis, atheroma formation, and tissue immunity [41]. Notably, IP-10 was shown to trigger the emergence of dormant breast cancer cells, making IP-10/CXCR3 as a potential target for breast cancer therapy [42,43]. In agreement with our results from MCF-7 breast carcinoma cells, IP-10 induction by TNF-α has been also reported in hepatocytes [20]. Choi et al. also reported that TNF-α induced CXCL10 in MDA-MB-231 breast cancer cells in NF-κB-dependent manner [44]. Of note, Mulligan et al. assessed expression of IP-10/ CXCL-10 and its receptor CXCR3 in 364 breast carcinomas using tissue microarrays and showed that IP-10 could play a role in lymphocytic infiltrate migration and invasion, suggesting that IP-10 acted in a paracrine manner to affect the tumor microenvironment as well as in an autocrine manner to act on the tumor cells themselves and thus played a role in tumor progression and invasiveness. This study reported that the level of expression of IP-10 was higher in breast cancer tumors of the basal subtype as compared to ER+ tumors and that IP-10/CXCR3 axis could serve as a potential target in BRCA1 and basal breast cancers that involve increased lymphocytic infiltration and a poor prognosis [45]. Similarly, increased expression/activation of other tumor microenvironment modulators, such as matrix metalloproteinase (MMP)-2 and MMP-9 in breast cancer, was found to be associated, at least partially, with the expression of HER)-2/neu in ER-positive disease and predicted malignancy and poor survival [46]. Our data may have implications with regard to interaction between breast tumor cells and their microenvironment, as the biologically relevant concentrations of TNF-α in the tumor microenvironment may lead to enhanced/sustained expression of IP-10 by the tumor cells.

Next, we showed that TNF-induced IP-10 production by MCF-7 breast cancer cells involves signaling via the JNK pathway as JNK inhibition by SP600125 or siRNA-mediated genetic silencing of JNK, or c-Jun attenuated the TNF-induced IP-10 production. Indeed, basal JNK activity is essential for the proliferation and maintenance of diploidy in breast cancer cells. JNK is a multi-factorial kinase implicated in several pathophysiological processes. Our data showing the JNK activation following TNF-α stimulation are aligned with other reports indicating activation of the JNK activity by various types of stress stimuli, including heat shock, UV light, protein synthesis inhibition, and mechanical injury [47,48] as well as by proinflammatory cytokines, such as TNF-α and IL-1, in different cell types [49,50,51]. Not surprisingly, the mammalian JNKs were originally named as stress-activated protein kinase (SAPK), as they were activated by various types of environmental stresses [52]; however, the JNK pathway was also found to respond to cell stimulations with growth factors or proinflammatory cytokines, such as TNF-α and IL-1 [53].

Although the effects of TNF-α stimulation, such as activation of NF-κB, in cancer and other cells are well known [54,55,56], the mechanism by which it upregulates activity of the AP-1-related transcription factors in MCF-7 cells remains unclear. We found that TNF-α stimulation enhanced the phosphorylation of c-Jun in MCF-7 cells. Furthermore, as expected, JNK kinase assay confirmed the c-Jun phosphorylation by TNF-α in MCF-7 cells. Indeed, the inflammatory response to TNF-α is mediated, at least in part, through regulation of gene expression by the transcription factors that are related to AP-1 and NF-κB [57]. The canonical and noncanonical pathways of NF-κB signal transduction are activated by direct engagement of the TNFR1 and CD40 receptors, respectively [58]; on the other hand, AP-1-related transcription factors are activated by an indirect mechanism involving MAPK [59]. JNK activation regulates the activity of c-Jun by phosphorylation and by activating transcription factors of the AP-1 family [60]. AP-1 activation is mediated by two distinct regulatory events: first, some AP-1 proteins are induced after TNF-α treatment during immediate-early gene transcription, and later, some AP-1 proteins are post-translationally modified to enhance the transcriptional activity. Collectively, ours and other studies support the role of JNK/c-Jun axis in TNF-α-induced IP-10 expression in breast cancer or other cells. Nonetheless, caution will be advised while interpreting these results, as our study is limited by certain caveats. First, the data presented are based on MCF-7 breast cancer cell line only, and it remains unclear whether similar mechanisms regulate the IP-10 expression in breast cancer cell lines other than MCF-7, such as T47D, or in metastatic (infiltrative)/ non-metastatic (in situ) breast cancer cells. Next, in addition to TNF-α, the changes in IFN-γ and its receptors (IFN-γ-Rα/β) are gaining wider interest due to their modulatory effects on cell growth/differentiation and inflammatory responses and also for being a potential therapeutic tool in breast cancer [61,62,63]. Therefore, it would be of further interest to see whether the co-stimulation with TNF-α and IFN-γ may have a synergistic effect on IP-10 induction in breast cancer and other cell types.

## 5. Conclusions

In conclusion, our data show that TNF-α stimulation induces the IP-10 transcripts/protein expression in MCF-7 breast cancer cells through the mechanism involving JNK/c-Jun-mediated signaling, which implies that targeting the JNK/c-Jun kinases of MAPK pathway may have significance in alleviating TNFα-/IP10-mediated breast cancer progression and metastasis.

## Data Availability

Not applicable.

## Supplementary Materials

The following are available online at https://www.mdpi.com/article/10.3390/biom11091355/s1, Figure S1: TNF-α-induced IP-10 expression in MDA-MB 231 breast cancer cells is suppressed by inhibiting MAPK signaling pathways. Figure S2: Effect of NF-kB inhibition on TNF-α induced IP-10 in MCF-7 cells.

## References

[1] Magesh P., Thankachan S., Venkatesh T., Suresh P.S. (2021). Breast cancer fibroblasts and cross-talk. Clin. Chim. Acta Int. J. Clin. Chem..

[2] Nagarsheth N., Wicha M.S., Zou W. (2017). Chemokines in the cancer microenvironment and their relevance in cancer immunotherapy. Nat. Rev. Immunol..

[3] Griffith J.W., Sokol C.L., Luster A.D. (2014). Chemokines and chemokine receptors: Positioning cells for host defense and immunity. Annu. Rev. Immunol..

[4] Luster A.D., Jhanwar S.C., Chaganti R.S., Kersey J.H., Ravetch J.V. (1987). Interferon-inducible gene maps to a chromosomal band associated with a (4;11) translocation in acute leukemia cells. Proc. Natl. Acad. Sci. USA.

[5] Chang C.-C., Wu C.-L., Su W.-W., Shih K.-L., Tarng D.-C., Chou C.-T., Chen T.-Y., Kor C.-T., Wu H.-M. (2015). Interferon gamma-induced protein 10 is associated with insulin resistance and incident diabetes in patients with nonalcoholic fatty liver disease. Sci. Rep..

[6] Qin S., Rottman J.B., Myers P., Kassam N., Weinblatt M., Loetscher M., Koch A.E., Moser B., Mackay C.R. (1998). The chemokine receptors CXCR3 and CCR5 mark subsets of T cells associated with certain inflammatory reactions. J. Clin. Investig..

[7] Hiratsuka I., Itoh M., Yamada H., Yamamoto K., Tomatsu E., Makino M., Hashimoto S., Suzuki A. (2015). Simultaneous measurement of serum chemokines in autoimmune thyroid diseases: Possible role of IP-10 in the inflammatory response. Endocr. J..

[8] Chalin A., Lefevre B., Devisme C., Pronier C., Carrière V., Thibault V., Amiot L., Samson M. (2018). Serum CXCL10, CXCL11, CXCL12, and CXCL14 chemokine patterns in patients with acute liver injury. Cytokine.

[9] Chen L.J., Lv J., Wen X.Y., Niu J.Q. (2013). CXC chemokine IP-10: A key actor in liver disease?. Hepatol. Int..

[10] Sørensen T.L., Trebst C., Kivisäkk P., Klaege K.L., Majmudar A., Ravid R., Lassmann H., Olsen D.B., Strieter R.M., Ransohoff R.M. (2002). Multiple sclerosis: A study of CXCL10 and CXCR3 co-localization in the inflamed central nervous system. J. Neuroimmunol..

[11] Sajadi S.M., Khoramdelazad H., Hassanshahi G., Rafatpanah H., Hosseini J., Mahmoodi M., Arababadi M.K., Derakhshan R., Hasheminasabzavareh R., Hosseini-Zijoud S.M. (2013). Plasma levels of CXCL1 (GRO-alpha) and CXCL10 (IP-10) are elevated in type 2 diabetic patients: Evidence for the involvement of inflammation and angiogenesis/angiostasis in this disease state. Clin. Lab..

[12] Strieter R.M., Kunkel S.L., Arenberg D.A., Burdick M.D., Polverini P.J. (1995). Interferon gamma-inducible protein 10 (IP-10), a member of the C-X-C chemokine family, is an inhibitor of angiogenesis. Biochem. Biophys. Res. Commun..

[13] Romagnani P., Lasagni L., Annunziato F., Serio M., Romagnani S. (2004). CXC chemokines: The regulatory link between inflammation and angiogenesis. Trends Immunol..

[14] Jabeen S., Espinoza J.A., Torland L.A., Zucknick M., Kumar S., Haakensen V.D., Lüders T., Engebraaten O., Børresen-Dale A.-L., Kyte J.A. (2018). Noninvasive profiling of serum cytokines in breast cancer patients and clinicopathological characteristics. Oncoimmunology.

[15] Yoshimatsu G., Kunnathodi F., Saravanan P.B., Shahbazov R., Chang C., Darden C.M., Zurawski S., Boyuk G., Kanak M.A., Levy M.F. (2017). Pancreatic β-Cell-Derived IP-10/CXCL10 Isletokine Mediates Early Loss of Graft Function in Islet Cell Transplantation. Diabetes.

[16] Ioannidis L.J., Eriksson E., Hansen D.S. (2020). CD14(+) monocytes are the main leucocytic sources of CXCL10 in response to Plasmodium falciparum. Parasitology.

[17] Lee J.H., Kim B., Jin W.J., Kim H.H., Ha H., Lee Z.H. (2017). Pathogenic roles of CXCL10 signaling through CXCR3 and TLR4 in macrophages and T cells: Relevance for arthritis. Arthritis Res. Ther..

[18] Ye J., Ma C., Wang F., Hsueh E.C., Toth K., Huang Y., Mo W., Liu S., Han B., Varvares M.A. (2013). Specific recruitment of γδ regulatory T cells in human breast cancer. Cancer Res..

[19] Alomar S.Y., Gentili A., Zaibi M.S., Kępczyńska M.A., Trayhurn P. (2016). IL-1β (interleukin-1β) stimulates the production and release of multiple cytokines and chemokines by human preadipocytes. Arch. Physiol. Biochem..

[20] Narumi S., Yoneyama H., Inadera H., Nishioji K., Itoh Y., Okanoue T., Matsushima K. (2000). TNF-alpha is a potent inducer for IFN-inducible protein-10 in hepatocytes and unaffected by GM-CSF in vivo, in contrast to IL-1beta and IFN-gamma. Cytokine.

[21] Lunardi S., Jamieson N.B., Lim S.Y., Griffiths K.L., Carvalho-Gaspar M., Al-Assar O., Yameen S., Carter R.C., McKay C.J., Spoletini G. (2014). IP-10/CXCL10 induction in human pancreatic cancer stroma influences lymphocytes recruitment and correlates with poor survival. Oncotarget.

[22] Al-Rashed F., Ahmad Z., Snider A.J., Thomas R., Kochumon S., Melhem M., Sindhu S., Obeid L.M., Al-Mulla F., Hannun Y.A. (2021). Ceramide kinase regulates TNF-alpha-induced immune responses in human monocytic cells. Sci. Rep..

[23] Al-Roub A., Akhter N., Al-Sayyar A., Wilson A., Thomas R., Kochumon S., Al-Rashed F., Al-Mulla F., Sindhu S., Ahmad R. (2021). Short Chain Fatty Acid Acetate Increases TNFalpha-Induced MCP-1 Production in Monocytic Cells via ACSL1/MAPK/NF-kappaB Axis. Int. J. Mol. Sci..

[24] Kochumon S., Al-Rashed F., Abu-Farha M., Devarajan S., Tuomilehto J., Ahmad R. (2019). Adipose tissue expression of CCL19 chemokine is positively associated with insulin resistance. Diabetes Metab. Res. Rev..

[25] Sindhu S., Kochumon S., Shenouda S., Wilson A., Al-Mulla F., Ahmad R. (2019). The Cooperative Induction of CCL4 in Human Monocytic Cells by TNF-alpha and Palmitate Requires MyD88 and Involves MAPK/NF-kappaB Signaling Pathways. Int. J. Mol. Sci..

[26] Al-Rashed F., Ahmad Z., Thomas R., Melhem M., Snider A.J., Obeid L.M., Al-Mulla F., Hannun Y.A., Ahmad R. (2020). Neutral sphingomyelinase 2 regulates inflammatory responses in monocytes/macrophages induced by TNF-α. Sci. Rep..

[27] Al-Rashed F., Sindhu S., Arefanian H., Al Madhoun A., Kochumon S., Thomas R., Al-Kandari S., Alghaith A., Jacob T., Al-Mulla F. (2020). Repetitive Intermittent Hyperglycemia Drives the M1 Polarization and Inflammatory Responses in THP-1 Macrophages Through the Mechanism Involving the TLR4-IRF5 Pathway. Cells.

[28] Kochumon S., Al Madhoun A., Al-Rashed F., Thomas R., Sindhu S., Al-Ozairi E., Al-Mulla F., Ahmad R. (2020). Elevated adipose tissue associated IL-2 expression in obesity correlates with metabolic inflammation and insulin resistance. Sci. Rep..

[29] Kochumon S., Arefanian H., Azim R., Shenouda S., Jacob T., Abu Khalaf N., Al-Rashed F., Hasan A., Sindhu S., Al-Mulla F. (2020). Stearic Acid and TNF-alpha Co-Operatively Potentiate MIP-1alpha Production in Monocytic Cells via MyD88 Independent TLR4/TBK/IRF3 Signaling Pathway. Biomedicines.

[30] Kochumon S., Wilson A., Chandy B., Shenouda S., Tuomilehto J., Sindhu S., Ahmad R. (2018). Palmitate Activates CCL4 Expression in Human Monocytic Cells via TLR4/MyD88 Dependent Activation of NF-κB/MAPK/ PI3K Signaling Systems. Cell. Physiol. Biochem..

[31] Sindhu S., Akhter N., Wilson A., Thomas R., Arefanian H., Al Madhoun A., Al-Mulla F., Ahmad R. (2020). MIP-1alpha Expression Induced by Co-Stimulation of Human Monocytic Cells with Palmitate and TNF-alpha Involves the TLR4-IRF3 Pathway and Is Amplified by Oxidative Stress. Cells.

[32] Thomas R., Al-Rashed F., Akhter N., Al-Mulla F., Ahmad R. (2019). ACSL1 Regulates TNFalpha-Induced GM-CSF Production by Breast Cancer MDA-MB-231 Cells. Biomolecules.

[33] Ahmad R., Al-Roub A., Kochumon S., Akther N., Thomas R., Kumari M., Koshy M.S., Tiss A., Hannun Y.A., Tuomilehto J. (2018). The Synergy between Palmitate and TNF-alpha for CCL2 Production Is Dependent on the TRIF/IRF3 Pathway: Implications for Metabolic Inflammation. J. Immunol..

[34] Al-Rashed F., Thomas R., Al-Roub A., Al-Mulla F., Ahmad R. (2020). LPS Induces GM-CSF Production by Breast Cancer MDA-MB-231 Cells via Long-Chain Acyl-CoA Synthetase 1. Molecules.

[35] Parameswaran N., Patial S. (2010). Tumor necrosis factor-α signaling in macrophages. Crit. Rev. Eukaryot. Gene Expr..

[36] Harris D.P., Bandyopadhyay S., Maxwell T.J., Willard B., DiCorleto P.E. (2014). Tumor necrosis factor (TNF)-alpha induction of CXCL10 in endothelial cells requires protein arginine methyltransferase 5 (PRMT5)-mediated nuclear factor (NF)-kappaB p65 methylation. J. Biol. Chem..

[37] Chin B.Y., Choi M.E., Burdick M.D., Strieter R.M., Risby T.H., Choi A.M. (1998). Induction of apoptosis by particulate matter: Role of TNF-alpha and MAPK. Am. J. Physiol..

[38] Zhao X.W., Zhou J.P., Bi Y.L., Wang J.Y., Yu R., Deng C., Wang W.K., Li X.Z., Huang R., Zhang J. (2019). The role of MAPK signaling pathway in formation of EMT in oral squamous carcinoma cells induced by TNF-α. Mol. Biol. Rep..

[39] Cruceriu D., Baldasici O., Balacescu O., Berindan-Neagoe I. (2020). The dual role of tumor necrosis factor-alpha (TNF-α) in breast cancer: Molecular insights and therapeutic approaches. Cell. Oncol..

[40] Zhou X.L., Fan W., Yang G., Yu M.X. (2014). The clinical significance of PR, ER, NF- kappa B, and TNF- alpha in breast cancer. Dis. Markers.

[41] Liu M., Guo S., Stiles J.K. (2011). The emerging role of CXCL10 in cancer (Review). Oncol. Lett..

[42] Clark A.M., Heusey H.L., Griffith L.G., Lauffenburger D.A., Wells A. (2021). IP-10 (CXCL10) Can Trigger Emergence of Dormant Breast Cancer Cells in a Metastatic Liver Microenvironment. Front. Oncol..

[43] Tokunaga R., Zhang W., Naseem M., Puccini A., Berger M.D., Soni S., McSkane M., Baba H., Lenz H.-J. (2018). CXCL9, CXCL10, CXCL11/CXCR3 axis for immune activation—A target for novel cancer therapy. Cancer Treat. Rev..

[44] Choi J., Ahn S.S., Lim Y., Lee Y.H., Shin S.Y. (2018). Inhibitory Effect of Alisma canaliculatum Ethanolic Extract on NF-κB-Dependent CXCR3 and CXCL10 Expression in TNFα-Exposed MDA-MB-231 Breast Cancer Cells. Int. J. Mol. Sci..

[45] Mulligan A.M., Raitman I., Feeley L., Pinnaduwage D., Nguyen L.T., O’Malley F.P., Ohashi P.S., Andrulis I.L. (2013). Tumoral lymphocytic infiltration and expression of the chemokine CXCL10 in breast cancers from the Ontario Familial Breast Cancer Registry. Clin. Cancer Res. Off. J. Am. Assoc. Cancer Res..

[46] Pellikainen J.M., Ropponen K.M., Kataja V.V., Kellokoski J.K., Eskelinen M.J., Kosma V.-M. (2004). Expression of Matrix Metalloproteinase (MMP)-2 and MMP-9 in Breast Cancer with a Special Reference to Activator Protein-2, HER2, and Prognosis. Clin. Cancer Res..

[47] Zhang P., Miller B.S., Rosenzweig S.A., Bhat N.R. (1996). Activation of C-jun N-terminal kinase/stress-activated protein kinase in primary glial cultures. J. Neurosci. Res..

[48] Pereira A.M., Tudor C., Kanger J.S., Subramaniam V., Martin-Blanco E. (2011). Integrin-dependent activation of the JNK signaling pathway by mechanical stress. PLoS ONE.

[49] Reinhard C., Shamoon B., Shyamala V., Williams L.T. (1997). Tumor necrosis factor alpha-induced activation of c-jun N-terminal kinase is mediated by TRAF2. EMBO J..

[50] Wicovsky A., Müller N., Daryab N., Marienfeld R., Kneitz C., Kavuri S., Leverkus M., Baumann B., Wajant H. (2007). Sustained JNK Activation in Response to Tumor Necrosis Factor Is Mediated by Caspases in a Cell Type-specific Manner. J. Biol. Chem..

[51] Tang N., Zhang Y.-P., Ying W., Yao X.-X. (2013). Interleukin-1β upregulates matrix metalloproteinase-13 gene expression via c-Jun N-terminal kinase and p38 MAPK pathways in rat hepatic stellate cells. Mol. Med. Rep..

[52] Johnson G.L., Nakamura K. (2007). The c-jun kinase/stress-activated pathway: Regulation, function and role in human disease. Biochim. Biophys. Acta.

[53] Hah Y.-S., Kang H.-G., Cho H.-Y., Shin S.-H., Kim U.-K., Park B.-W., Lee S.-I., Rho G.-J., Kim J.-R., Byun J.-H. (2013). JNK signaling plays an important role in the effects of TNF-α and IL-1β on in vitro osteoblastic differentiation of cultured human periosteal-derived cells. Mol. Biol. Rep..

[54] Schütze S., Wiegmann K., Machleidt T., Krönke M. (1995). TNF-induced activation of NF-kappa B. Immunobiology.

[55] McDonald P.P., Bald A., Cassatella M.A. (1997). Activation of the NF-κB Pathway by Inflammatory Stimuli in Human Neutrophils. Blood.

[56] Cheng X., Shi W., Zhao C., Zhang D., Liang P., Wang G., Lu L. (2016). Triptolide sensitizes human breast cancer cells to tumor necrosis factor-α-induced apoptosis by inhibiting activation of the nuclear factor-κB pathway. Mol. Med. Rep..

[57] Baud V., Karin M. (2001). Signal transduction by tumor necrosis factor and its relatives. Trends Cell Biol..

[58] Hayden M.S., Ghosh S. (2014). Regulation of NF-κB by TNF family cytokines. Semin. Immunol..

[59] Ventura J.-J., Kennedy N.J., Lamb J.A., Flavell R.A., Davis R.J. (2003). c-Jun NH(2)-terminal kinase is essential for the regulation of AP-1 by tumor necrosis factor. Mol. Cell. Biol..

[60] Whitmarsh A.J., Davis R.J. (1996). Transcription factor AP-1 regulation by mitogen-activated protein kinase signal transduction pathways. J. Mol. Med..

[61] García-Tuñón I., Ricote M., Ruiz A.A., Fraile B., Paniagua R., Royuela M. (2007). Influence of IFN-gamma and its receptors in human breast cancer. BMC Cancer.

[62] Yaghoobi H., Azizi H., Oskooei V.K., Taheri M., Ghafouri-Fard S. (2018). Assessment of expression of interferon γ (IFN-G) gene and its antisense (IFNG-AS1) in breast cancer. World J. Surg. Oncol..

[63] Wang L., Simons D.L., Lu X., Tu T.Y., Avalos C., Chang A.Y., Dirbas F.M., Yim J.H., Waisman J., Lee P.P. (2020). Breast cancer induces systemic immune changes on cytokine signaling in peripheral blood monocytes and lymphocytes. EBioMedicine.

