# Supplementary material for: TNF-α Increases IP-10 Expression in MCF-7 Breast Cancer Cells via Activation of the JNK/c-Jun Pathways
Source: Biomolecules. 2021 Sep 13;11(9):1355. doi: 10.3390/biom11091355 (PMC8464892; doi:10.3390/biom11091355)
Supplement: Supplementary file 1 [file biomolecules-11-01355-s001.zip › biomolecules-1339654-supplementary.pdf]

Supplementary Data File

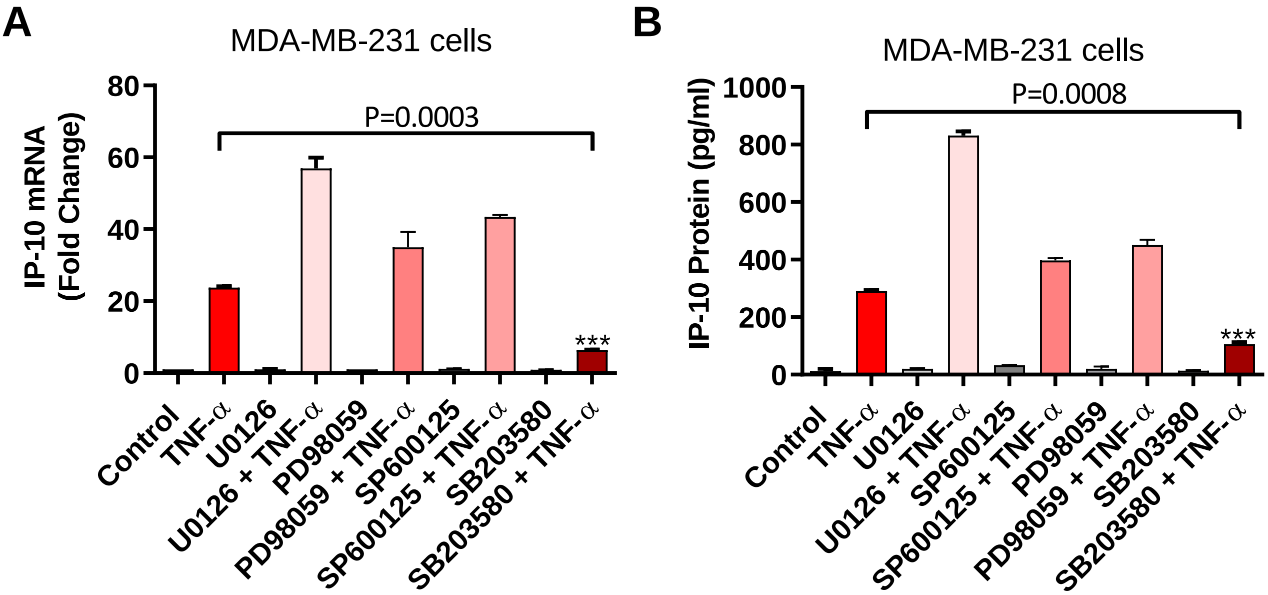

Figure S1. TNF-α-induced IP-10 expression in MDA-MB 231 breast cancer cells is suppressed by inhibiting MAPK signaling pathways. (A and B). MDA-MB 231 breast cells were pretreated for 1 h with inhibitors of MEK1/2 (U0126; 10 µM), ERK1/2 (PD98059; µM), JNK (SP600125; 10 µM), p38 (SB203580; 10 µM) and then incubated with TNF-α for 24 hrs. Cells and culture media were collected. IP-10 mRNA and protein expression were determined using real-time RT-PCR and ELISA, respectively. . All data are expressed as mean ± SEM (n = 3). *** $p < 0.001$.

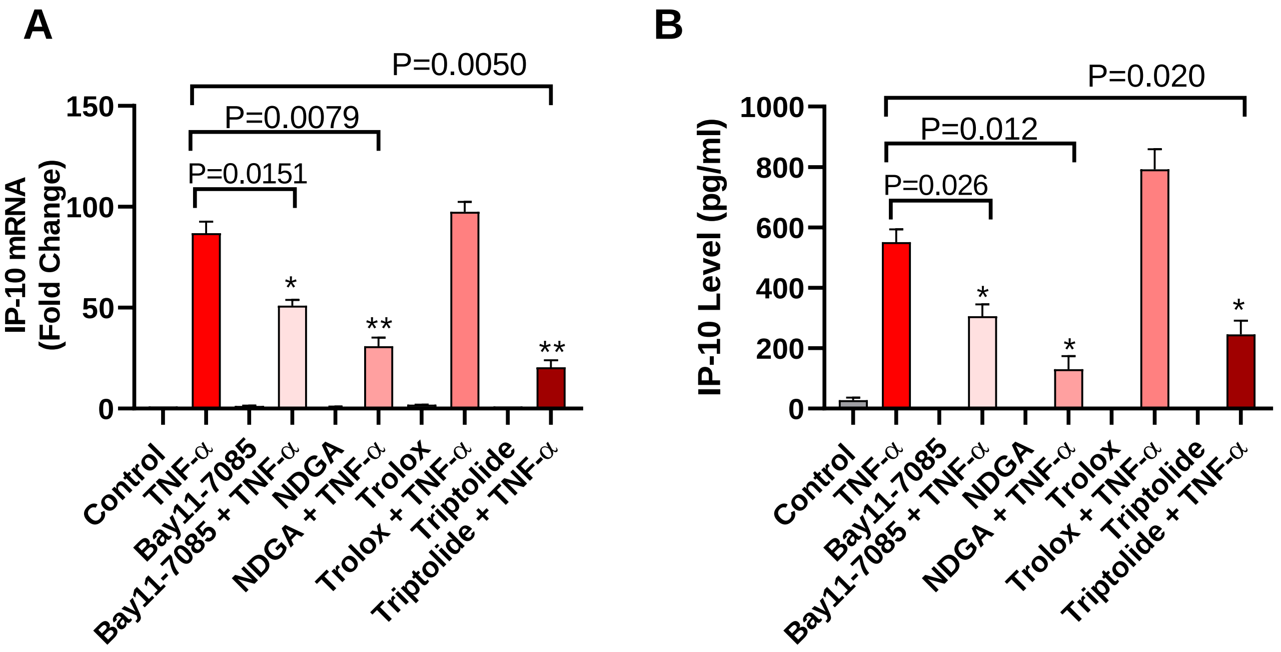

Figure S2. Effect of NF-kB inhibition on TNF-α induced IP-10 in MCF-7 cells. (A-B) MCF-7 cells were pretreated with NF-κB inhibitors (Bay11-7085, 10 uM; NDGA, 30 uM; Trolox, 10 μM; Triptolide, 10 μM) for 1 h and then treated with TNFα (10 ng/mL) for 24 h. Cells and culture media were collected. IP-10 mRNA and protein expression were determined using real-time RT-PCR and ELISA, respectively. All data are expressed as mean ± SEM (n = 3). * $p < 0.05$, ** $p < 0.01$.
